# An Improved Method of Measuring Wavefront Aberration Based on Image with Machine Learning in Free Space Optical Communication

**DOI:** 10.3390/s19173665

**Published:** 2019-08-23

**Authors:** Yangjie Xu, Dong He, Qiang Wang, Hongyang Guo, Qing Li, Zongliang Xie, Yongmei Huang

**Affiliations:** 1 Institute of Optics and Electronics, Chinese Academy of Sciences, No.1 Guangdian Road, Chengdu 610209, China; 2 Key Laboratory of Optical Engineering, Chinese Academy of Sciences, Chengdu 610209, China; 3 University of Chinese Academy of Sciences, Beijing 100049, China; 4 School of Information and Communication Engineering, University of Electronic Science and Technology of China, No.2006 Xiyuan Ave, West Hi-Tech Zone, Chengdu 611731, China

**Keywords:** free space optical communication, phase retrieval, machine learning

## Abstract

In this paper, an improved method of measuring wavefront aberration based on image with machine learning is proposed. This method had better real-time performance and higher estimation accuracy in free space optical communication in cases of strong atmospheric turbulence. We demonstrated that the network we optimized could use the point spread functions (PSFs) at a defocused plane to calculate the corresponding Zernike coefficients accurately. The computation time of the network was about 6–7 ms and the root-mean-square (RMS) wavefront error (WFE) between reconstruction and input was, on average, within 0.1263 waves in the situation of D/r0 = 20 in simulation, where D was the telescope diameter and r0 was the atmospheric coherent length. Adequate simulations and experiments were carried out to indicate the effectiveness and accuracy of the proposed method.

## 1. Introduction

Wavefront aberrations generated by atmospheric turbulence affect the distribution of focus, then deteriorate the fiber coupling efficiency and the quality of communication. Measurement of wavefront aberration in free space optical communication differs from other scenarios in that the atmospheric turbulence changes constantly. Correction of wavefront aberration is based on the correlation of each frame, so real-time performance determines the performance of the correction. High-accuracy correction of wavefront aberration requires accurate real-time measurement in free space optical communication. The methods of measuring wavefront aberration are mainly divided into two classes. The first method measures wavefront aberration by monitoring the wavefront slope, which needs additional wavefront sensors, such as a Hartmann sensor or interferometer [1,2,3]. The other method uses the distribution of focus as the objective function and optimizes the objective function with continuous iteration, such as an image-based sensor [4,5,6,7]. The real-time performance of this second method is poor and so its application range is very limited.

Owing to the high fitting ability and successful application of machine learning in other fields, some research on measuring wavefront aberration with machine learning has been completed. A back propagation (BP) neural network was used to measure wavefront aberration and was verified on the Hubble telescope [8,9,10]. The input to the network is a one-dimensional vector which is composed by all pixels of the point spread functions (PSFs) in the focal and defocus planes. Through a series of matrix operations and activation functions, the network outputs the Zernike coefficients. The BP neural network has some disadvantages, such as poor generalization ability and getting local optimal solution.

In order to improve the generalization ability of the aforementioned network, research groups used Tchebichef moment as the input of the network [11,12]. The addition of Tchebichef moment allows the network to process PSFs of different sizes. Furthermore, a deep neural network was used to replace the BP neural network because of its better fitting ability. As a result, the root-mean-square (RMS) error between the target and output of the network in the testing set was found to be 0.0089 waves (4th~9th Zernike coefficients corresponding to focus, astigmatism, coma and spherical aberration). The high accuracy proved that two PSFs can provide enough information to measure low-order wavefront aberration. However, the calculation of image moments requires significant time [13], which was not consistent with our original intention for using machine learning.

In recent years, convolutional neural networks (CNNs) have emerged, which, when using a convolution filter, are more suitable for processing images compared to BP neural networks and deep neural networks [14,15]. Inception V3 [16], a convolutional neural network that performs well in image classification, was used to measure wavefront aberration in Reference [17]. In this study, the input to the network was the PSF in the focal plane and the output of the network was the initial estimate of the Zernike coefficients. The output of the network was, on average, within 0.37 waves RMS wavefront error (WFE) of the true solution. Using these initial estimations as the starting value of the nonlinear optimization, the error was reduced to within 0.1 waves. The addition of Inception V3 shortened the nonlinear optimization process that originally required 16 s to 0.2 s. However, the accuracy of the network was not high enough to eliminate nonlinear optimization.

Some preconditioners, such as overexposure, defocus, and scatter, have been used to improve the accuracy of the network [18]. Experimental results show that this method is very effective. However, researchers have only considered the situation of weak turbulence and have not mentioned the real-time performance.

In order to advance the real-time performance and fitting accuracy of measuring wavefront aberration with a CNN, an improved method is proposed in this paper. We demonstrated that the trained network could be used to calculate Zernike coefficients without nonlinear optimization for the case of strong atmospheric turbulence. The PSF in the defocus plane replaced the PSF in the focal plane as the input to the network, because the latter would lead to a multi-solution problem. A new convolutional neural network we optimized was used as a training model. Some adaptations of the network made obvious improvements to the fitting accuracy. In particular, the addition of a batch normalization (BN) layer improved the ability of the network. The output of our network was Zernike coefficients. Note that this output can be replaced with other parameters in a specific system, such as the control voltage of the deformation mirror, which may have better fitting precision and take less time. However, the generalization ability of the network will be weakened. Adequate simulations and experiments were carried out to indicate the effectiveness and accuracy of the proposed method for the case of strong atmospheric turbulence.

This article consists of five chapters. In Section 1, we give a brief introduction to the application of machine learning in measuring atmospheric turbulence. In Section 2, the method is presented. The simulation and results are presented in Section 3 and Section 4. In Section 5, we summarize the article.

## 2. Method

### 2.1. Imaging System

Based on the imaging principle, the image on the focal plane CCD can be written as:

(1) I(x,y)=o(x,y)⊗PSF(x,y)

where *I(x, y)* is the image of the focal CCD, *o(x, y)* is the ideal intensity distribution, and ⊗ is a convolution operation. *PSF(x, y)* is the point spread function of the system, which can be written as:

(2) $$PSF\left(x,y\right)={\left|ℱ\left(w\right)\right|}^{2}$$

(3) $$w\left(r,\theta \right)=\overset{n}{\underset{i=1}{\sum }}a_{i}z_{i}\left(r,\theta \right)$$

where ℱ is the Fourier transform, *w* is the wavefront, $a_{i}$ are Zernike coefficients and $z_{i}$ are Zernike polynomials. *r* is the radial distance and *θ* is the azimuthal angle in polar coordinates. The simulated random wavefronts follow the Kolmogorov turbulence model [19].

When the image plane is on the focal plane, the image calculation in the defocus plane can be equivalent to adding an additional phase to wavefront, wherein the additional phase ∆φ can be written as:

(4) $$∆\varphi =-\frac{k\times d}{2\times \left(f+d\right)\times f}\times \left(u^{2}+v^{2}\right)$$

where *d* is the focal shift, f is the focal length, and *u* and *v* are the coordinates of the pupil plane. k=2π/λ is wave vector.

### 2.2. Structure of the CNNs

**Figure 1:**
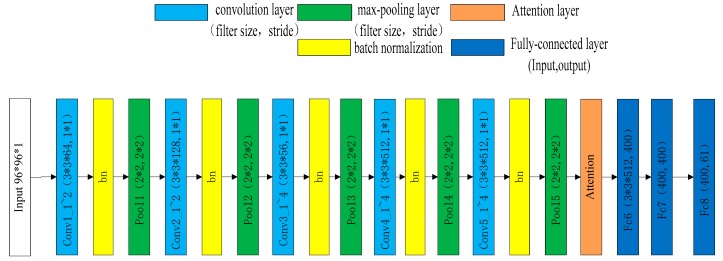
Structure of convolutional neural network3 (CNN3). Conv1_1~2 means conv1_1 and conv1_2.

The CNNs optimized by us were CNN1, CNN2 and CNN3. The CNN3 we optimized was composed of a convolution layer filter, a max-pooling layer filter, a rectified linear unit (*ReLU)*, a fully-connected layer, a batch normalization layer filter and an attention layer, as shown in Figure 1. Compared with CNN3, CNN2 excluded the attention layer, while CNN1 excluded both the BN layer and the attention layer. The learning algorithm used was Adam [20] and the learning rate was 0.0001. The loss function was the RMS difference between the predicted Zernike coefficients and the true Zernike coefficients.

#### 2.2.1. Batch Normalization Layer Filter

The batch normalization layer filter played an important role in the network. Convolutional neural networks often present a gradient-disappearing problem during training. The usual method to deal with this problem is to train layer by layer. However, in this work, a batch normalization layer filter solved this problem. Specifically, the batch normalization layer filter converted the input of this layer into a normal distribution with a mean of 0 and a variance of 1. Most of the data was then transferred to the central area, where the gradient was usually the largest or was present. This effectively prevented the appearance of the vanishing gradient problem. Importantly, the batch normalization layer filter can also speed up training, increase accuracy, and reduce over-fitting. The batch normalization layer filter can be written as [21]:

(5) $${\hat{x}}_{i}=\;\frac{x_{i}-E\left(x_{i}\right)}{\sqrt{Var\left(x_{i}\right)}}$$

(6) $$y_{i}\;=\;\gamma {\hat{x}}_{i}+\beta$$

where $x_{i}$ is the *i*-th input of the mini-batch (the sample processed for each iteration). ${\hat{x}}_{i}$ is the normalized value of $x_{i}$. $E\left(x_{i}\right)=\frac{1}{m}\sum_{i=1}^{m}x_{i}$ is the mean of the mini-batch. $Var\left(x_{i}\right)=\frac{1}{m}\sum_{i=1}^{m}{\left(x_{i}-E\left(x_{i}\right)\right)}^{2}$ is the variance of the mini-batch. $y_{i}$ is the output of batch normalization layer filter. γ and β are the mean and variance of the all elements of the feature map, respectively, which make the network learn to recover the distribution of features that the original network has to learn.

#### 2.2.2. Attention Layer

The attention layer consisted of both channel-wise attention and spatial attention. The channel-wise attention modulated the weight of the channels, helping the network find the feature maps which had more information. The spatial attention modulated the attention weight of space, which meant different pixels had different weight. The pixels which had more information had more weight. The model of channel-wise attention can be written as [22]:

(7) $$a=tanh\left(Wc⊗v+b_{c}\right)$$

(8) α=softmax(a)

where v =${\left[\upsilon_{1},\upsilon_{2},\ldots ,\upsilon_{C}\right]}^{T}$, *C* is the total number of channels, and $\upsilon_{i}$ is the mean of the *i*-th channel of the feature map. $Wc\in {\mathbb{R}}^{c*c}$ are transformation matrices and $b_{c}$ are bias terms. Softmax is $\alpha_{i}=\;e^{a_{i}}/\left(\sum_{j=1}^{c}e^{a_{j}}\right)$ and α is the weight of channel-wise attention.

The model of spatial attention can be written as:

(9) $$b=tanh\left(Ws⊗V+b_{s}\right)$$

(10) β=softmax(b)

where we reshape $V=\left[v_{1},v_{2},\ldots ,v_{m}\right]$ by flattening the width and height of feature map, $V_{1}\in {\mathbb{R}}^{c}$, m=W×H, and H is the width and height of feature map. $Ws\in {\mathbb{R}}^{1c}$ are transformation matrixs and $b_{s}$ is bias terms. β is the weight of spatial attention.

## 3. Simulation

In this section, we describe the validation of the proposed method through simulations, including feasibility verification, sample size impact and generalization ability.

### 3.1. Feasibility Verification

**Figure 2:**
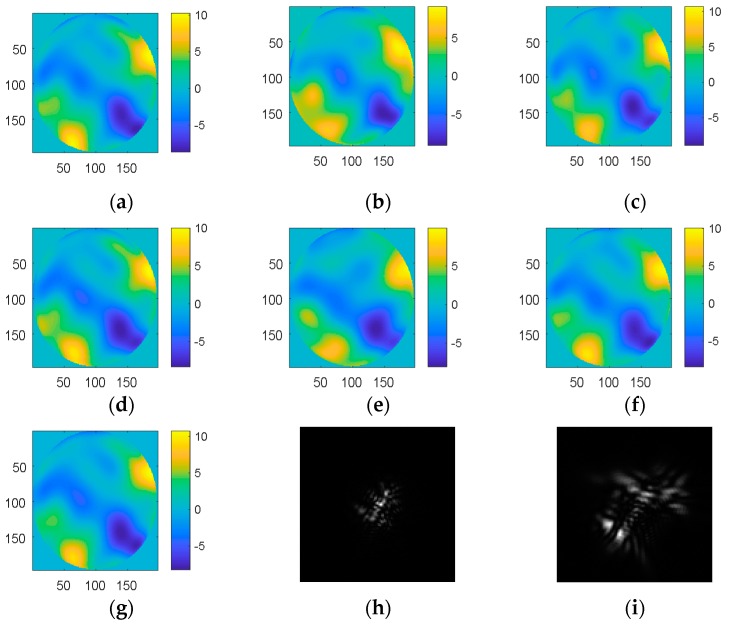
The wavefront of 4th to 64th Zernike coefficients. (**a**) The simulated wavefront; (**b**–**g**) the wavefront of reconstructions by Alexnet, VGG, Inception V3, CNN1, CNN2, and CNN3; (**h**) the focal plane point spread functions (PSF); and (**i**) the defocused PSF.

Firstly, 22,000 samples, which were simulated combining 4th to 64th Zernike polynomials with D/r0 = 20 and wavelength 850 nm, were used to improve the CNN’s ability to measure aberrations. The training set consisted of 20,000 samples and the testing set was another 2000 samples. The 1st (global piston), 2nd (tip), and 3rd (tilt) Zernike coefficients were not included because they can be easily measured by centroiding algorithms. The RMS of the input wavefront was 0.6789λ. The true and reconstructed wavefront are shown in Figure 2. The mean root-mean-squares (MRMSs) of the WFEs after correction are shown in Table 1. The algorithm was run on a desktop PC with a 32 GB DDR4 RAM and a 1080ti GPU.

The results showed that the wavefront of the reconstructions were similar to the true wavefronts when the defocused PSF was used as the input to the network. The mean root-mean-square (MRMS) of the wavefront error met the requirements in most cases. Inception V3 had the highest estimation accuracy, but it also had the longest computation time. Inception V3 had better real-time performance compared to other networks in terms of classification, but a decrease in the output of other networks reduced their computation time because they used a fully-connected layer. Thus, other networks performed better in terms of time as the output of these networks consisted of only 61 parameters.

Our optimized network had a good real-time performance because of the decrease in input size. However, this degraded the result. For this reason, a BN layer and an attention layer were added to the network. Upon comparing the results from CNN1 and CNN2 with CNN3, it was noted that the addition of a BN layer noticeably improved the accuracy, while an attention layer improved the accuracy even further. Compared with other networks, the CNN3 we optimized had good real-time performance and estimation accuracy.

**Figure 3:**
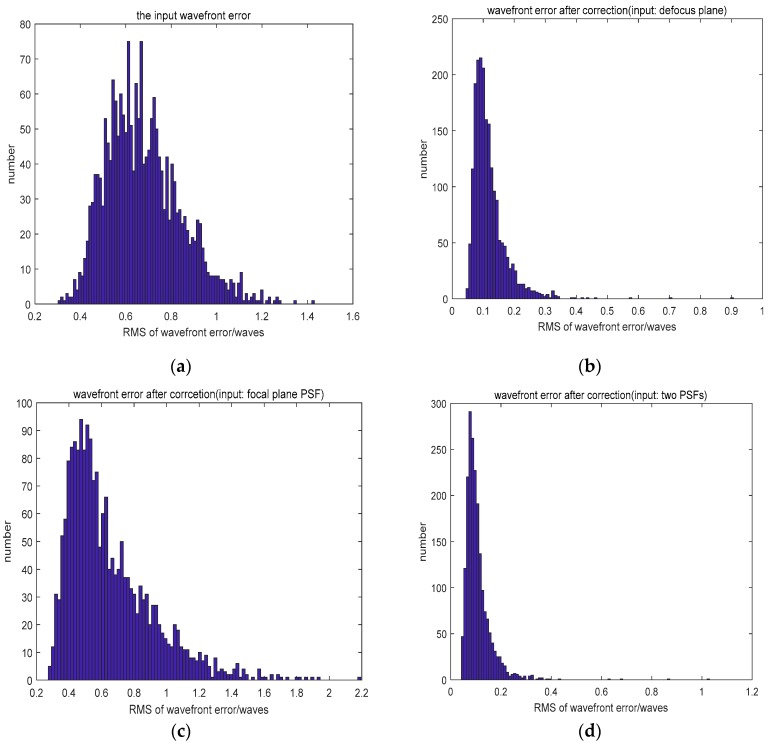
(**a**) The distribution of the input wavefront error and (**b**–**d**) the distribution of wavefront error after correction.

Compared to the networks which employed an input of defocused PSF, using the focal plane PSF as the input did not decrease the wavefront error because of its multi-solution problem. Although the use of two PSFs (the focal plane PSF and defocused PSF) as the input resulted in a similar fitting accuracy, it took more time and required more calculations. The distribution of wavefront errors is shown in Figure 3.

### 3.2. Simulations with Different Sample Sizes

Next, in order to determine the number of PSFs that needed to be collected in the experiment, the impact of different sample sizes on wavefront error was tested. Specifically, 5000 PSFs, 10,000 PSFs, 15,000 PSFs and 20,000 PSFs were used as the training sets and 2000 PSFs was used as the testing set. From the 20,000 PSFs, 5000 PSFs, 10,000 PSFs and 15,000 PSFs were selected at random. The testing set underwent the same selection, thereby minimizing the influence of the content of the sample. The samples were simulated combining 4th to 64th Zernike polynomials with D/r0 = 20 and wavelength 850 nm. The results are shown in Table 2.

The MRMS of the wavefront error decreased when the sample size increased. As 0.16λ is usually used as the standard of correction, 20,000 PSFs were collected for training in the experiment. The simulation results also indicated that the wavefront error could be decreased if more samples were used to train.

### 3.3. Generalization Ability

The trained networks’ performance in measuring wavefront aberration when the atmospheric coherence length (r0) was different needed to be proven. PSFs which were simulated combining 4th to 64th Zernike polynomials when D/r0 = 6, 10, and 15 were used as the testing set to test the networks which were trained with the PSFs simulated by Zernike coefficients when D/r0 = 20. The RMS of true WFE and the MRMS of WFE after correction are shown in Table 3.

The simulations proved that most networks had good generalization ability. The trained network could measure wavefront aberrations corresponding to different atmospheric coherent lengths. In addition, the MRMS of the wavefront error decreased because the atmospheric turbulence was weaker when D/r0 decreased. This was consistent with our perception. However, Inception V3′s generalization ability seemed to be worse.

The simulations showed that, compared with other networks, CNN3 had good real-time performance, high estimation accuracy and generalization ability.

## 4. Experiment

**Figure 4:**
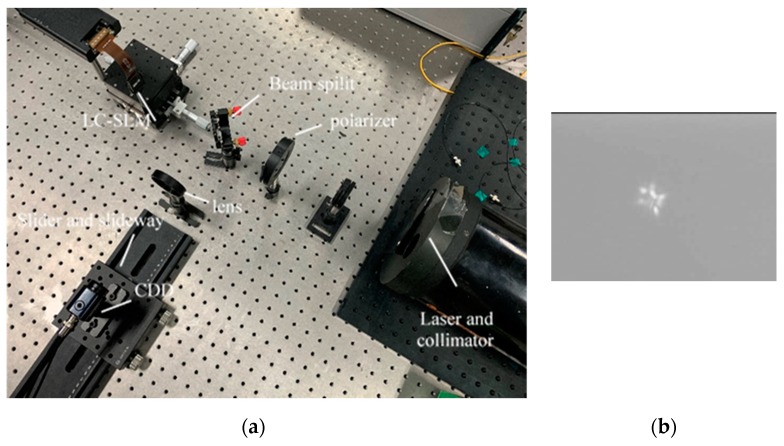
(**a**) The system used in the experiment and (**b**) the PSF of the defocused plane.

The experimental platform is shown in Figure 4. The light source employed was a laser with wavelength 850 nm and an LC-SLM (PLUTO-2-NIR-011, Holoeye, pixel pitch: 8 µm, pixel count: 1920 × 1080) was used as a spatial modulator to simulate atmospheric turbulence. A polarizer was added since the LC-SLM employed acts on P-polarized light. The CCD was placed on a rail in order to access defocused PSFs. The atmospheric turbulence situation was D/r0 = 6.

**Figure 5:**
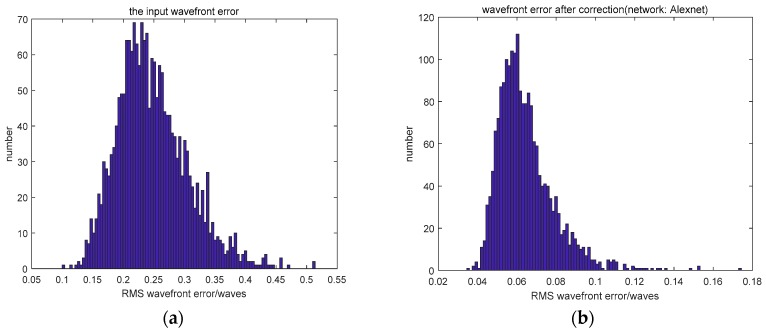

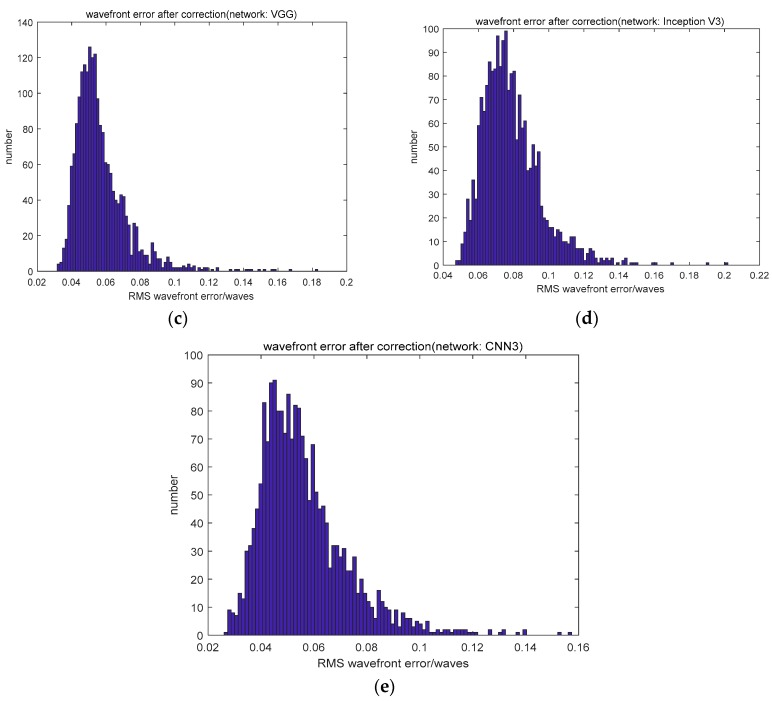
(**a**) The distribution of the input wavefront error and (**b**–**e**) the distribution of the wavefront error after correction.

We collected 22,000 defocused PSFs in the experiment. Of these, 20,000 PSFs formed the training set and the other 2000 PSFs were the testing set. The results of the experiment are shown in Table 4. The MRMS of the input wavefront was 0.2463λ. The distribution of wavefront error is shown in Figure 5.

The results of the experiment show that the networks can use the defocused PSF to calculate the corresponding Zernike coefficients accurately. CNN3 had similar estimation accuracy to other networks. However, CNN3 took less time for computation. We found that the experimental results were better than the simulation results, as the testing set of the experiment was the same situation as the training set. In contrast, the testing set of the simulation (D/r0 = 6) was different to the training set used to train the network (D/r0 = 20).

In summary, CNN3 can used to measured wavefront aberration and perform well in fitting accuracy. Although other networks have similar fitting accuracy, CNN3′s computation time is less. As the correction of wavefront aberration is based on the correlation of each frame image, CNN3 is more suitable for practical applications.

## 5. Conclusions

In this paper, an improved method of measuring wavefront aberration based on image intensity was described and validated with an experiment. The proposed method had good real-time performance and high estimation accuracy. We demonstrated that the trained network could be used to calculate Zernike coefficients accurately. The root-mean-square wavefront error of CNN3 between reconstruction and input was, on average, within 0.1263 waves when D/r0 = 20 (simulation) and 0.0521 waves when D/r0 = 6 (experiment). After inputting an image, CNN3 only took 6–7 ms to output Zernike coefficients. Thus, the computation time of the network decreased a lot and the network performed well.

Our future work will focus on building a closed-loop real-time system, which can be used in free space optical communication. We will test the performance of such networks with dynamic aberrations.

## Figures and Tables

**Table 1:** The wavefront errors of the networks.

Number	Training Set	Network	MRMS of WFE (Testing Set)	Computation Time
1	defocused PSF	Alexnet	0.2183λ	6.5–7.5 ms
2	defocused PSF	VGG	0.1490λ	11–12 ms
3	defocused PSF	Inception V3	0.1187λ	24–28 ms
4	defocused PSF	CNN1	0.2371λ	5–6 ms
5	defocused PSF	CNN2	0.1344λ	6–7 ms
6	defocused PSF	CNN3	0.1263λ	6–7 ms
7	focal PSF	CNN3	0.6510λ	6–7 ms
8	two PSFs	CNN3	0.1248λ	7–8.5 ms

λ = 850 nm.

**Table 2:** The results of the simulations using different sample sizes.

Number	Training Set	Network	MRMS of WFE (Testing Set)
1	5000 PSFs	CNN3	0.2255λ
2	10,000 PSFs	CNN3	0.1597λ
3	15,000 PSFs	CNN3	0.1360λ
4	20,000 PSFs	CNN3	0.1263λ

λ = 850 nm.

**Table 3:** The simulation of generalization ability.

Networks	MRMS of WFE (D/r0 = 6)	MRMS of WFE (D/r0 = 10)	MRMS of WFE (D/r0 = 15)	MRMS of WFE (D/r0 = 20)
Input	0.2463λ	0.3780λ	0.5272λ	0.6789λ
Alexnet	0.1145λ	0.1220λ	0.1732λ	0.2183λ
VGG	0.0884λ	0.0981λ	0.1122λ	0.1490λ
Inception V3	0.1360λ	0.0965λ	0.0922λ	0.1187λ
CNN1	0.1286λ	0.1273λ	0.1681λ	0.2371λ
CNN2	0.0754λ	0.0709λ	0.0962λ	0.1344λ
CNN3	0.0705λ	0.0629λ	0.0833λ	0.1263λ

λ = 850 nm.

**Table 4:** The results of the experiment.

No.	Network	MRMS of WFE (Testing Set)	Computation Time
1	Alexnet	0.0625λ	6.5–7.5 ms
2	VGG	0.0578λ	11–12 ms
3	Inception V3	0.0782λ	24–28 ms
4	CNN3	0.0521λ	6–7 ms

λ = 850 nm.
